# Birth-and-Death Evolution and Reticulation of ITS Segments of *Metschnikowia andauensis* and *Metschnikowia fructicola* rDNA Repeats

**DOI:** 10.3389/fmicb.2018.01193

**Published:** 2018-06-12

**Authors:** Matthias Sipiczki, Eniko Horvath, Walter P. Pfliegler

**Affiliations:** Department of Genetics and Applied Microbiology, University of Debrecen, Debrecen, Hungary

**Keywords:** ribosomal RNA, ITS, evolution, pseudogene, reticulation, hybridisation, yeast, structure prediction

## Abstract

The internal transcribed spacer (ITS) region (ITS1, 5.8S rDNA, and ITS2) separates the genes coding for the SSU 18S and the LSU 26S genes in the rDNA units which are organized into long tandem arrays in the overwhelming majority of fungi. As members of a multigenic family, these units are subject of concerted evolution, which homogenizes their sequences. Exceptions have been observed in certain groups of plants and in a few fungal species. In our previous study we described exceptionally high degree of sequence diversity in the D1/D2 domains of two pulcherrimin-producing *Metschnikowia* (Saccharomycotina) species which appeared to evolve by reticulation. The major goals of this study were the examination of the diversity of the ITS segments and their evolution. We show that the ITS sequences of these species are not homogenized either, differ from each other by up to 38 substitutions and indels which have dramatic effects on the predicted secondary structures of the transcripts. The high intragenomic diversity makes the D1/D2 domains and the ITS spacers unsuitable for barcoding of these species and therefore the taxonomic position of strains previously assigned to them needs revision. By analyzing the genome sequence of the *M. fructicola* type strain, we also show that the rDNA of this species is fragmented, contains pseudogenes and thus evolves by the birth-and-death mechanism rather than by homogenisation, which is unusual in yeasts. The results of the network analysis of the sequences further indicate that the ITS regions are also involved in reticulation. *M. andauensis* and *M. fructicola* can form interspecies hybrids and their hybrids segregate, providing thus possibilities for reticulation of the rDNA repeats.

## Introduction

The internal transcribed spacers ITS1 and ITS2 of the ribosomal DNA (rDNA) cistrons separate genes coding for ribosomal RNAs which are essential components of ribosomes. The rDNA cistrons are repeated many times in the genome accross the tree of life, so that enough rRNA can be produced when demand for ribosomes is high. In most species for which relevant molecular data are available, the rDNA cistrons flanked by characteristic intergenic regions are organized as tandem head-to-tail repeats (rDNA repeats) in continuous arrays (for a review see Torres-Machorro et al., 2010). In spite of the large number of repeats, nucleotide polymorphism within the rDNA array is usually very low (e.g., Ganley and Kobayashi, 2007). By a process called “concerted evolution” (Zimmer et al., 1980; Elder and Turner, 1995), the repeats within the arrays are maintained essentially identical (homogenized) even over evolutionarily significant timescales. It ensures that a mutation that arises in one repeat is either eliminated or spreads by inter-copy interactions through the array until fixation.

**FIGURE 1:**
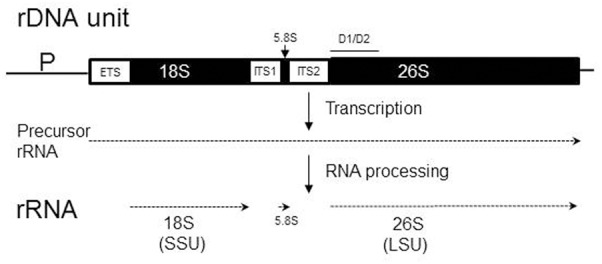
Organization and transcription of the chromosomal rDNA unit (repeat) containing ribosomal RNA (rRNA) genes. The three rRNA genes (18S, 5.8S, and 26S) are shown as solid boxes, while regions removed from the precursor transcript during RNA maturation (ETS, external transcribed spacer; ITS, internal transcribed spacer) are open boxes. The 18S rRNA is integrated into the small subunit (SSU) of the ribosome, whereas the 5.8S and 26S rRNAs are embedded into the large subunit (LSU). P, promoter. The position of the D1/D2 domain is shown by the line over the 26S rRNA gene.

The ITS spacers are transcribed together with the rRNA genes of the cistron but their RNA copies are not incorporated into mature ribosomes, but removed from the ribosomal precursor RNA (prerRNA) by a specific cleavage process that is catalyzed by the secondary structures of the ITS transcripts themselves (for reviews, see Venema and Tollervey, 1999; Coleman, 2015) (**Figure 1**). Despite this activity, these sequences are assumed to evolve neutrally (Schlotterer et al., 1994) and thus considered suitable as genomic markers for taxonomic identification and phylogenetic reconstructions (Hillis and Dixon, 1991). Since their sequence similarity is greater within a species than between species (barcoding gap), the ITS region was recently designated the official barcode for fungi (Schoch et al., 2012). In our previous work (Sipiczki et al., 2013) we presented data indicating that the rDNA homogenisation mechanism is not always effective. We demonstrated that two *Metschnikowia* species had non-homogenized pools of D1/D2 domains of LSU (26S) rRNA genes.

The ascomycetous yeast genus *Metschnikowia* consists of over 80 species (Lachance, 2016). The small-spored *M. pulcherrima* and five related species (*M. andauensis, M. fructicola, M. shanxiensis, M. sinensis*, and *M. ziziphicola*) produce pulcherrimin, a red-brownish pigment in their cells and also externally, in the medium, observable as halos around the colonies growing on agar media (e.g., Kluyver et al., 1953; Sipiczki, 2006; Xue et al., 2006; Lachance, 2011). Pulcherrimin is a water-insoluble complex of Fe^3+^ ions and the diffusible pulcherriminic acid (a derivative of the cyclo-L-leucyl-L-leucyl dipeptide) (Cook and Slater, 1956; Cryle et al., 2010). The depletion of the medium of free Fe^3+^ by the formation of the pigment has inhibitory effect on the germination of fungal conidia and the growth of certain molds, yeasts and bacteria (Sipiczki, 2006). A recent phylogenetic analysis based on data from four protein-coding genes placed the pulcherrimine-producing species on a lineage clearly separated from the rest of the genus (Guzmán et al., 2013).

We found that the type strains of *M. andauensis* and *M. fructicola* have non-homogenized rDNA arrays (Sipiczki et al., 2013). The intragenomic diversity of the D1/D2 domains of the large subunit (LSU; 26S) rDNA (**Figure 1**) hampers the taxonomic separation of them from each other and from related species by D1/D2 sequencing. Both strains turned out to have D1/D2 units of diverse sequences that do not form distinct clades on the phylogenetic trees and networks and appear to evolve by reticulation including interspecies interactions. The aims of this study were to examine the diversity of ITS segments of the rDNA repeats of these species by cloning and sequencing individual paralogs, the identification of ITS sequences in the draft genome sequence of the *M. fructicola* type strain and their phylogenetic analysis. We show that the ITS sequences of *M. andauensis* and *M. fructicola* are not homogenized either and are likewise placed intermixed in the phylogenetic networks. Hence, neither D1/D1 nor ITS sequencing can be used for barcoding of these species. By analyzing the genome sequence of the *M. fructicola* type strain, we also show that the rDNA of this species is fragmented, contains pseudogenes and thus evolves by the birth and death mechanism rather than by homogenisation. In fungi, birth-and-death mode of rDNA evolution was observed only in the case of dispersed 5S genes (Rooney and Ward, 2005) which, however, have no relevance for barcoding and taxonomic identification. We also present data demonstrating that both species can form interspecies hybrids and the hybrids segregate, providing thus possibilities for reticulation of their rDNA repeats with those of related species.

## Materials and Methods

### Strains and Culture Conditions

The *M. andauensis* and *M. fructicola* strains used are listed in Supplementary Table S1. The composition of the growth media YEA (yeast-extract agar), YEL (yeast-extract liquid), and SMA (synthetic minimal agar), were described previously (Sipiczki, 2012). Sporulation was tested on V-8 agar (Pitt and Miller, 1968) and vegetable juice (BIO Gemüsesaft, Josef Pölz, Bio-Produkte, 84518 Garching an der Alz, Germany) agar. V8 juice was filtered with filter paper, diluted 1:20 with distilled water, adjusted to pH 5.5 by NaOH, and the medium thus prepared autoclaved. The vegetable juice was used in 20x dilution without pH adjustment. Both media contained 2% agar.

### Amplification, Cloning, and Sequencing of ITS1-5.8S-ITS2

Nuclear DNA was isolated from overnight cultures grown in YEL broth as described previously (Sipiczki, 2003). The isolated DNA was used for the amplification of the ITS1-5.8S-ITS2 regions of the rRNA repeats with the primers ITS1 and ITS4 (White et al., 1990) and GoTaq polymerase (Promega, Madison, United States). The amplified DNA was used either for direct sequencing or for random cloning of individual ITS1-5.8S-ITS2 fragments into the pGEM-T Easy Vector, following the manufacturer’s instructions (Promega, Madison, United States). In the latter case, bacterial colonies were randomly selected from the transformants. Plasmids were extracted from the colonies and checked for the size of the inserts by reamplification with the same primer pair. The amplified DNA was sequenced in both directions using the same primers. The sequences were deposited in GenBank under accession numbers listed in Supplementary Table S2. The cloned sequences were compared with each other using the bl2seq algorithm available in NCBI^1^. Search for similar database sequences was conducted using the blastn services of the NCBI Blast web site and the UNITE database^2^.

### Search for ITS Sequences in the *M. fructicola* Genome Sequence

The only sequence found in more than one clones was used as query sequence to find complete and partial ITS1-5.8S-ITS2 segments in the draft genome sequence of CBS 8853^T^, the type strain of *M. fructicola* available under the accession number ANFW00000000.2 in the NCBI Genome database^3^. The identified rDNA units were checked for completeness by the examination of their components, including the sequences of the genes coding for the 18S SSU (small subunit) and the 26S LSU (large subunit) rRNAs. All genomic ITS1-5.8S-ITS2 sequences were compared with each other and with the cloned sequences using bl2seq (see above).

### RNA Secondary Structure Prediction

ITS1 secondary structures were predicted from nucleotide sequences with the algorithm available at the mfold Web Server^4^ (Zuker, 2003). The delta G required for the formation of the secondary structures shown in relevant figure were -1.03 (Ifr9), -2.11 (Ifrc26), -3.10 (Ian10), -4.50 (Ifrc4), and -6.70 (Ian51). For the ITS2 consensus secondary structure prediction, the ITS2 database pipeline^5^ (Ankenbrand et al., 2015) was used. This workbench determines complete individual secondary structures for ITS2 sequences based on energy minimization (Markham and Zuker, 2008) or iterative homology modeling (Wolf et al., 2005). The secondary structures were re-drawn for publication purposes using the forna package http://rna.tbi.univie.ac.at/forna/ (Kerpedjiev et al., 2015). RNA weblogo was created using https://rth.dk/resources/slogo/ (Gorodkin et al., 1997).

### Phylogenetic and Network Analysis

To obtain multiple alignments for phylogenetic analyses, the sequences were aligned with the Clustal W 1.7 (Thompson et al., 1994) algorithm. Statistical parsimony networks were constructed with TCS 1.21, a program that implements the estimation of genealogies of DNA sequences from their multiple alignment (Clement et al., 2000). The default 95% cutoff was used. From the same Clustal alignments rooted rectangular phylogenetic networks and neighbor-net splits graphs were also created with the SplitsTree4 V4.12.8 package (Bryant and Moulton, 2004). In these analyses, the DNA evolution model HKY 85 (Hasegawa et al., 1985) was used for distance calculation. The outgroup of the rectangular phylogram was the sequence FJ623593 of *M. chrysoperlae* CBS 9803^T^. To generate neighbor-nets, Equal Angle setting was chosen and the sequence used as outgroup on the rectangular phylogram was excluded from the analysis, as the neighbor nets are unrooted networks. To test the aligned sequences for recombination, we used the Phi test of Bruen et al. (2006) as available in the Splits Tree4 package.

### Examination of Sporulation and Spore Germination

To induce sporulation, dense suspensions of overnight YPGL cultures were dropped on V-8 agar and vegetable juice agar. Sporulation was examined microscopically after incubation at 17°C at regular time intervals for 4 weeks. To visualize the nuclei of the ascospores, samples of the sporulating cultures were suspended in 40% ethanol, stained with 4′,6-diamidino-2-phenylindole (DAPI) (Sipiczki et al., 1998) and examined in a fluorescence microscope. Spore germination was examined microscopically in asci pulled out from dense suspensions of sporulating cultures with a micromanipulator on the surface of YEA plates and in samples of the cultures spread on thin YEA films prepared on glass slides and covered with cover slips. The latter (“sandwich”) method originally developed for the examination of morphological transitions in yeast cultures was described previously (Sipiczki et al., 1998). The incubation temperature was 20°C. Microphotographs were taken with an Olympus BX51 microscope and a DP70 digital camera. As the asci remained intact on the complete medium even after 4 weeks of incubation, we assumed that the failure of the ascus wall dissolution might prevent the spores from germination. Therefore we performed the experiments also with ascospores released from asci by Zymolyase 20T (MP Biomedicals, California, United States) treatment (0.5–1.0 mg/ml μg ml^-1^, 25°C, 3 h). To test the effect of freezing shock on germination activity, samples frozen to -20°C for 10 days before spreading on the test medium were also examined.

### Mutagenesis and Characterisation of Mutants

Cells of overnight cultures grown in YEL broth at room temperature were mutagenised either by UV irradiation on YEA plates or by culturing in YEL supplemented with nitrosoguanidine. For UV mutagenesis 10^3^ cells were spread on the plates and irradiated for time periods required to kill 85–90% of cells. After 7 days of incubation at 30°C, the colonies produced by the surviving cells were replica-plated onto SMA plates to identify auxotrophic mutants. The colonies that did not grow on SMA were isolated for physiology tests. To induce mutations by nitrosoguanidine, the 0.5-ml samples of the overnight culture (∼10^8^ cells ml^-1^) were added to 0.5 ml of fresh YEL supplemented with various amounts of nitrosoguanidine and incubated at room temperature for 1 h to allow DNA synthesis in the presence of the mutagen. The mutagenised cultures were then diluted with sterile water to ∼10^4^ cells ml^-1^ and 100-μl aliquots were spread on YEA plates. The colonies of plates showing 10–20% survival were replica-plated after 7 days of incubation onto SMA plates. The colonies whose replicas did not grow on SMA were isolated as putative auxotrophic mutants.

The nutritional requirements of the mutants were determined by the two-step disk method described previously (Sipiczki and Ferenczy, 1978). Briefly, two SMA plates were seeded with dense suspensions of cells of the mutant. Then six sterile filter-paper disks, each dipped into a different solution of 5–6 amino acids and nucleotides were placed in a hexagonal pattern on the surface of one plate. After 3 days of incubation, a zone of growth appeared around the disk that contained the compound for which the mutant was auxotrophic. Next, disks soaked separately with solutions of the individual members of the positive group were placed on the second plate. After 3 days of incubation, growth was visible around the disk soaked with the amino acid or nucleotide base which could not be synthesized in the mutant cells. The mutants are listed in Supplementary Table S1. The mutants used successfully in the hybridisation experiments have been deposited in the CBS culture collection (Centraalbureau voor Schimmelcultures, Utrecht, Netherlands).

### Hybridisation and Segregation Analysis

The auxotrophic mutants to be hybridized were streaked on YEA plates (two strains on one plate). After 3 days of incubation the line-shaped cultures were replica-plated onto fresh plates perpendicularly to each other to produce grids of prints in which the mutants intersected each other. After 7 days of incubation at 20°C, the “grids” were replica-plated onto SMA plates on which the mutants could not grow. If hybridization took place, prototrophic colonies appeared at the intersections. Individual colonies were isolated from the intersections and inoculated onto fresh SMA plates to obtain pure hybrid cultures.

To examine segregation, the hybrids were inoculated on vegetable agar plates and incubated at 13–15°C. After 4 weeks of incubation samples were taken from the cultures and spread on YEA plates (1) directly or (2) after Zymolyase treatment which lysed most vegetative cells and freed the ascospores from the asci or (3) after 10 days of freezing (-20°C). The colonies produced were replica-plated on SMA plates to identify auxotrophic segregants. The colonies that did not grow on SMA were isolated from the plates and tested for auxotrophies by inoculating them onto SMA plates supplemented with compounds for which the parental strains were auxotrophic.

## Results

### Diversity of Cloned ITS Sequences

When the DNA amplified with the ITS1 and ITS4 primers from the type strains was directly sequenced with the ITS1 primer, the chromatograms were not readable in the segments corresponding to the ITS1 spacers, and the sequences of the 5.8S and ITS2 parts contained about 20% ambiguous nucleotides. When sequenced from the ITS4 primer, the latter two segments had only a few ambiguous positions but ITS1 was chaotic again. These results implied that the ITS regions of the rDNA units like their D1/D2 domains (Sipiczki et al., 2013) were heterogeneous in both strains and thus unsuitable for their taxonomic differentiation. To verify the heterogeneity, individual molecules (6 from the *M. andauensis* type strain and 7 from the *M. fructicola* type strain) were cloned from the amplified DNA and sequenced individually. None of the cloned sequences had ambiguous positions but they turned out to differ from each other at numerous positions (**Table 1**). Both the ITS1 and ITS2 segments were much shorter than those of *S. cerevisiae* S288c^6^ and comparable in size to those of other *Metschnikowia* species. All but two ITS1 segments consisted of 74 nucleotides; the exceptions were Ian10 (73 nt) and Ian51 (76 nt). All ITS2 segments of both strains were 110 nucleotide long. 5 out of the 8 *M. fructicola* clones had identical sequences (Ifr4, Ifr6, Ifrb13, Ifrb15, and Ifrb17), whereas all *M. andauensis* clones had unique sequences. The most different sequences were *M andauensis* Ian51 and *M. fructicola* Ifrc26, but their difference (26 substitutions and 1 indel) was only slightly bigger than the biggest intraspecies differences (26 in *M. andauensis*) and (24 in *M. fructicola*).

**Table 1:** Number of differences in the pair-wise Blast alignments of the cloned sequences.

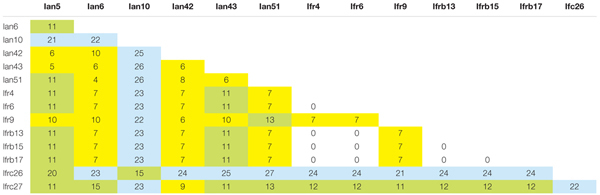

*Ian, ITS clone from the *M. andauensis* type strain; Ifr, ITS clone from the *M. fructicola* type strain White, no difference; yellow, 1–10 differences; green, 11–20 differences; blue, 21–27 differences.*

ITS1 was more diverse than ITS2. In the Clustal alignment of all cloned sequences 34 positions were variable in the ITS1 segments and four positions showed polymorphism in the ITS2 segments. Five *M. fructicola* clones and one *M. andauensis* clone had identical ITS2 sequences. In the 5.8S genes only one site was polymorphic. At this position all *M. andauensis* and two *M. fructicola* sequences had a cytosine whereas the rest of the *M. fructicola* sequences had a thymine. Interestingly, the range of intragenomic differences was broader in both strains than the differences of certain cloned sequences from the ITS sequences of the type strains of certain related species. For example Ian10 differed from Ian51 at 26 positions but only at 12 positions from KY104196 of *M. chrysoperlae* CBS 9803^T^. The ITS1 region of Ian51 differed at 10 sites from that of *M. rubicola* NRRL Y-6064^T^ (not released yet; Kurtzman et al., 2018) and at 14 positions from MF446617 of *M. persimmonesis* KIOM G15050^T^. The ITS1 region of Ifrb17 differed from that of Ifrc26 by 24 substitutions but only by 3 substitutions from the corresponding sequence of *M. leonuri* NRRL Y-6546^T^ (not released yet; Kurtzman et al., 2018). The blast search in the UNITE database identified a *M. bicuspidata* var. *bicuspidata* sequence (KP132407) which differed by 3 and 6 substitutions from Ifrb17 and Ian51, respectively.

### ITS Regions in a *M. fructicola* Draft Genome Sequence

**FIGURE 2:**
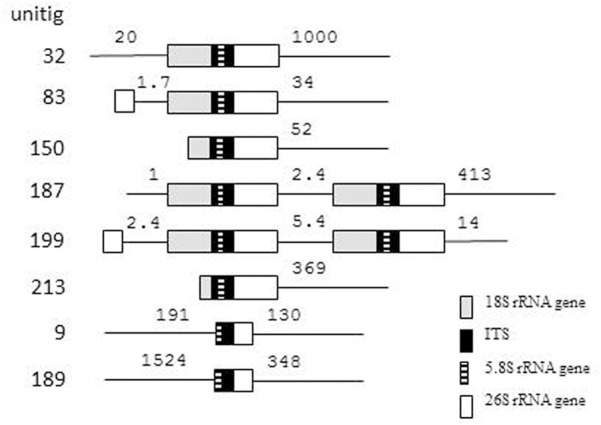
rDNA units in the genome sequence of *M. fructicola* CBS 8853^T^. The numbers show length in kb.

Recently the draft genome sequence of the strain *M. fructicola* 277 has been deposited in the NCBI genome database (Hershkovitz et al., 2013). This strain is presumably identical with the type strain of the species maintained in culture collections under collection numbers NRRL Y-27328^T^ and CBS 8853^T^ (Kurtzman and Droby, 2001). As we cloned the sequences from a CBS 8853^T^ culture, we searched ANFW01000000 for sequences similar to the clones. The blast search identified 8 loci containing full-length ITS1-5.8S-ITS2 regions (**Table 2** and **Figure 2**) and 2 incomplete copies. The sequence of the 5 identical *M. fructicola* clones was found only in the locus identified in unitig 150. None of the other loci showed 100% identity with any of the cloned sequences. When compared with each other, only the two genomic loci of unitig 187 showed 100% sequence identity. The pairwise differences of the rest were comparable to those found between the cloned sequences. The truncate copies in unitigs 9 and 189 lacked the entire ITS1 segment and their ITS2 sequences had 6 polymorphic sites that were conserved in the cloned sequences. Their 5.8S genes were very different from those in the cloned sequences and in the other genomic loci. The big difference between these truncate genomic regions and the cloned sequences shown in **Table 2** are mainly due to substitutions in the 5.8S genes.

**Table 2:** Number of differences in the pair-wise Blast alignments of the cloned sequences and the genomic loci.

Unitig	Unitig length	Position of			Difference (number of substitutions + indels) from									
			
		18S	ITS1-5.8S-ITS2	26S	Ifr4	Ifr9	Ifrc26	Ifrc27	Ian5	Ian6	Ian10	Ian42	Ian43	Ian51
					Ifr6									
					Ifrb13									
					Ifrb15									
					Ifrb17									
32	1,026,203	20,064–21,749	21,749–22,104	22,105–25,024	27	28	21	20	22	28	12	27	26	26
83	42,806	37,375–39,060	37,375–37,022	34,101–37,021 41,435–42,806	7	4	17	8	12	10	19	8	12	12
150	54,215	1–1,458	1,458–1,811	1,812–4,733	0	7	24	12	11	7	22	7	11	7
187	416,322	406,502–408,187	406,502–406,150	403,230–406,149	12	9	17	11	11	6	19	11	8	10
		413,854–415,539	413,794–413,502	410,582–413,501	12	9	17	11	11	6	19	11	8	10
199	29,094	2,785–4,469	4,469–4,820	4,821–7,738	5	4	22	11	10	7	20	6	10	9
		10,201–11,885	11,885–12,235	12,236–15,153	8	5	20	9	11	10	19	7	11	12
				1–316										
213	372,239	372,041–372,235	371,687–372,040	368,765–371,686	13	6	24	16	12	8	24	10	8	8
9^a^	324,760		191,058–191,333	191,336–191,540	26	26	26	25	24	26	26	23	24	23
189^a^	1,873,306		1,524,670–1,524,947	1,524,948–1,525,147	27	27	27	26	25	26	26	24	25	24


*^*a*^ITS1 is missing and the 5.8S sequence is very different from those in the cloned sequences.*

Two unitigs contained pairs of complete rDNA units. In 5 unitigs the units were terminally located indicating that they can be terminal parts of genomic rDNA arrays, which the assembling program could not fully reconstruct. The unit in unitig 32 is far enough from both ends of the unitig to assume that it might be a solo copy. The truncate copies were also internally located in their unitigs but were not accompanied by 18S genes and the 26S genes behind their ITS2 sequences were incomplete.

The complete 26S genes found in 8 loci offered a possibility to examine whether any of them had sequences identical with any of the D1/D2 domains cloned in our previous study (Sipiczki et al., 2013). Therefore we blasted the cloned D1/D2 sequences against ANFW01000000. Identity was found only between the *M. fructicola* D1/D2 clone fb6 (KC411964) and the 26S gene located in unitig 150. Remarkably, this rDNA unit had the only ITS sequence that was found also in certain cloned ITS segments. The rest of the genomic sequences differed by 1–11 and 2–15 substitutions from the D1/D2 clones of *M. fructicola* and *M. andauensis*, respectively. Similar search in *M. andauensis* could not be performed because its genome has not been sequenced yet.

### Phylogenetic Network Analysis

**FIGURE 3:**
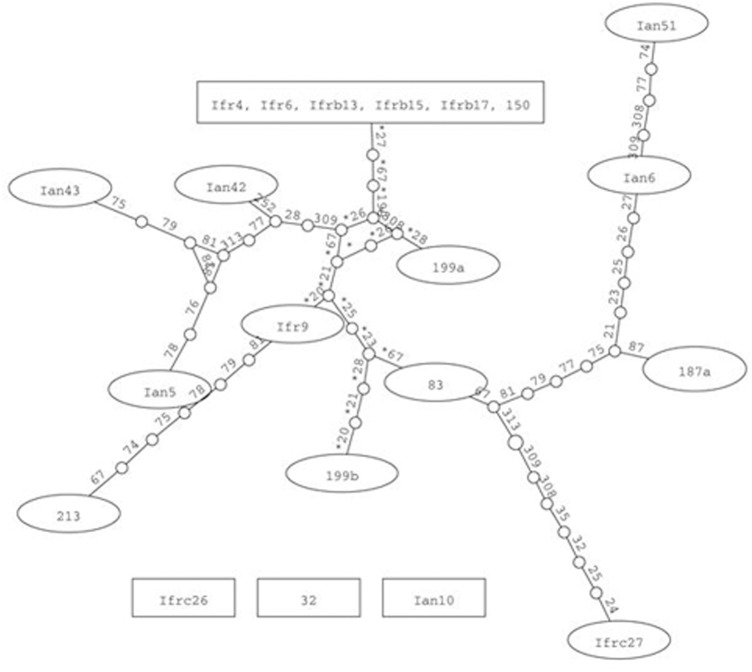
Parsimony network analysis showing the genealogical relationships of the cloned ITS1-5.8S-ITS2 sequences. The connections are based on the set of plausible solutions with a 95% of parsimony probability. Each connecting line represents one substitution and each small circle represents a missing intermediate sequence. The numerals between the circles are the serial numbers of nucleotides from the beginning of the alignment. A rectangle denotes the sequence identified as ancestral by the analysis. Ifr4 represents the group of five *M. fructicola* clones of identical sequences. Three sequences were excluded from the network by the algorithm.

Although we cannot exclude that certain genomic ITS segments could be sequence chimeras arising from misassembly of reads, we aligned them with the cloned sequences for phylogenetic network analyses. The statistical parsimony network analysis grouped most sequences of both type strains in a single network (**Figure 3**). Within the network, the sequences of the two strains did not form distinct lineages. This finding implies that the ITS sequences of the two strains might have joint evolutionary history, during which the rDNA units of the strains interacted many times. The program identified as ancestral the group of identical sequences cloned from *M. fructicola*. These sequences differed from certain *M. andauensis* members of the network by fewer substitutions than from certain other *M. fructicola* sequences. One *M. andauensis* clone, one *M. fructicola* clone and one genomic sequence were not connected to the network as if they had evolved from different ancestors.

**FIGURE 4:**
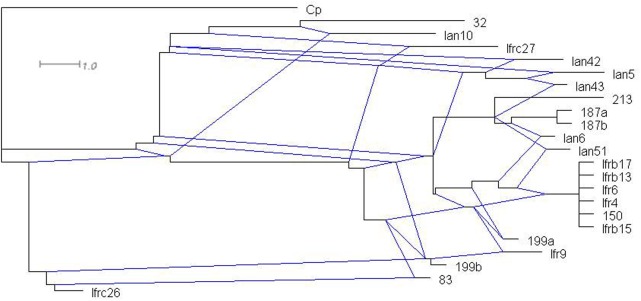
Rooted rectangular phylogenetic network of the cloned sequences. Cp (*C. picachoensis* CBS 9804^T^ AY494780) is the outgroup. The scale bar represents the split support for the edges.

**FIGURE 5:**
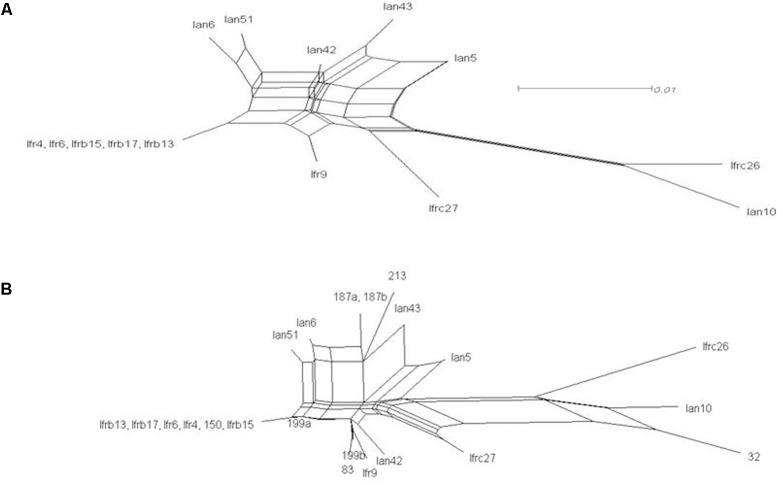
Network analysis of the cloned ITS1-5.8S-ITS2 sequences. Neighbor-net splits graphs. **(A)** Cloned sequences. **(B)** Cloned and genomic sequences. The scale bar represents the split support for the edges.

The intermixing of the sequences of the two strains suggested that their ITS regions evolved in a reticulate way. To visualize reticulate events we generated rooted rectangular phylogenetic networks and neighbor-net splits graphs with SplitsTree4 V4.12.8 (Bryant and Moulton, 2004). In the rooted rectangular phylogenetic network shown in **Figure 4**, the network edges connecting nodes in a non-bifurcational way indicate interactions between lineages. The high number of network edges clearly demonstrate that reticulation have played a major role in the evolution of the ITS segments examined. The result of the neighbor-net network analysis is shown in **Figure 5**. In a splits graph, a pair of nodes may be linked by a single edge (tree-like part) or a set of parallel edges. The latter depict alternative evolutionary possibilities (reticulate part). The box-like structures confirmed the notion that reticulation has occurred in the evolution of these sequences indeed. Reticulation involves complex processes such as horizontal transfer and exchange of genetic elements, hybridisation and recombination. The Phi test (Bruen et al., 2006) found statistically significant evidence for recombination (*p* = 2.784 E-8).

### Location of the Variable Positions and the Effect of Substitutions on the Predicted Secondary Structures of the Transcripts

**FIGURE 6:**
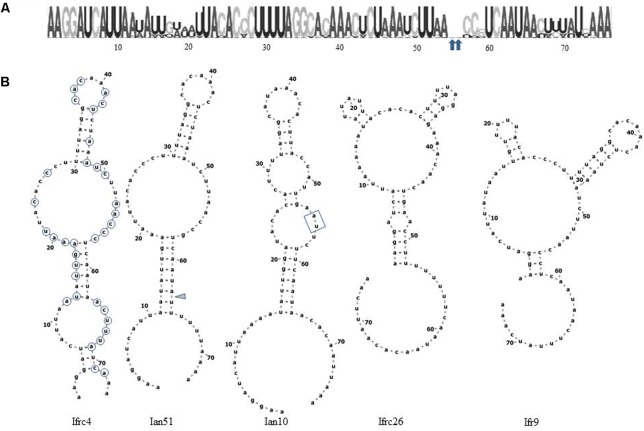
Structural analysis of ITS1. **(A)** RNA weblogo. Arrowheads denote sites of insertions of two nucleotides in Ian10. **(B)** Predicted secondary structures of cloned ITS1 sequences. The positions circled on Ifrc4 are variable. The insertions in Ian10 are boxed. The gray triangle marks the site of deletion in Ian51.

**FIGURE 7:**
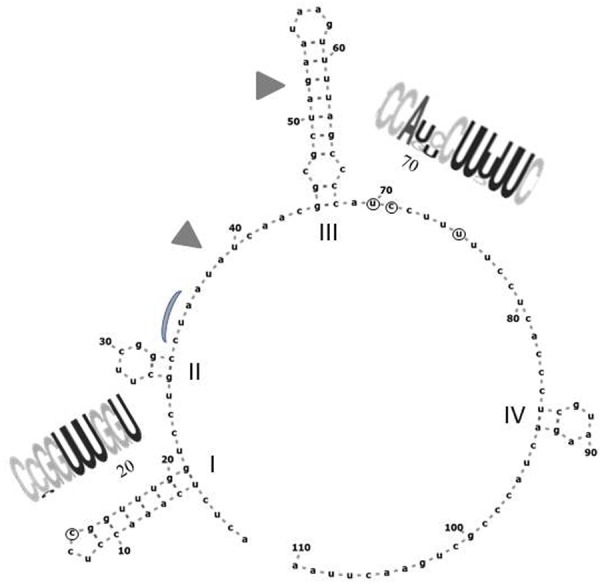
Predicted secondary structure of the ITS2 sequence of Ifrc4. Positions denoted with circes are variable in the cloned sequences. The conserved loops are labeled with Roman numerals. RNA weblogos show the sequences of the segments containing variable positions. Gray triangles mark the segments corresponding to the cut sites in *S. cerevisiae* (Coleman, 2015). The position corresponding to the binding site of the Nop15 protein involved in the processing of ITS2 in *S. cerevisiae* (Granneman et al., 2011) is marked with a gray curve.

The CLUSTAL alignment of all cloned sequences revealed that most nucleotides of the ITS1 and ITS2 regions were stable in all sequences, and the variable positions were not randomly scattered. To examine the possible structural effects of nucleotide differences in the variable positions, we generated secondary ITS1 and ITS2 rRNA structures from each cloned sequence. Secondary structures can provide information not found in the primary sequence (Caetano-Anolle’s, 2002). The ITS1 structures were very diverse (examples are presented in **Figure 6**) showing limited structural conservatism. Most but not all sequences formed a hairpin with several bulges of diverse size. The tip loop of these hairpins contained a conserved a-a pair at sites 39–40. These nucleotides may be important for the folding of the RNA transcript because they are conserved in the tip loop of the folded *S. cerevisiae* ITS1 as well (Coleman, 2015). The other variable positions formed four groups, three of them located in the backfolding stretch of the loop stem. Changes of nucleotides in certain positions caused drastic changes in the secondary structures. The ITS2 sequences had only four variable sites and their predicted secondary structures were identical. All had four loops with helix III as the longest loop (**Figure 7**). This structure corresponds to the core structure deduced from the analysis of 5000 ITS2 sequences (Schultz et al., 2005). In contrast to the substitution of ITS1, the substitutions in the ITS2 sequences had no effect on the predicted secondary structure. All but one variable positions grouped in the unpaired segment located between the Large loop III and the small loop IV. The only exception was in the tip of loop II.

### Hybridisation

**FIGURE 8:**
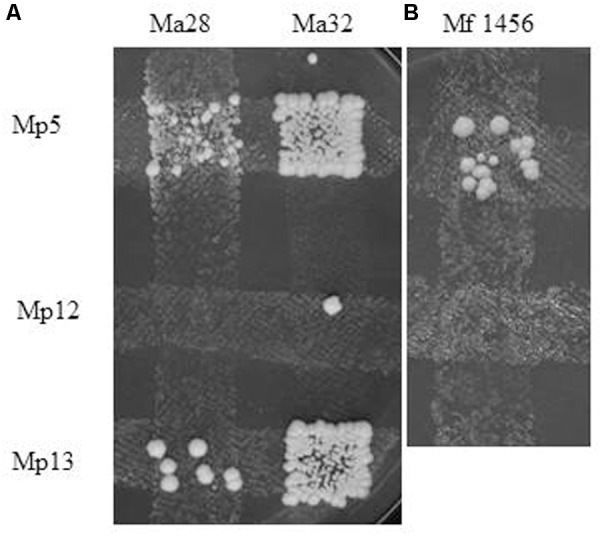
Interspecies hybridisation. Formation of prototrophs at the intersections of *M. pulcherrima* mutants with **(A)** *M. andauensis* and **(B)** *M. fructicola* mutants of opposite mating type and different auxotrophies. Ma32 is slightly leaky, the two colonies formed outside the intersections with Mp5 and Mp13 can be revertants. The week background growth in the intersection of Ma28 and Mp5 is due to crossfeeding of the former by the latter.

The biological isolation of the two strains was tested by hybridisation attempts. For easier identification of the hybrids, auxotrophic mutants were produced from both strains by mutagenesis. The stable mutants of different auxotrophies were mass-mated in all possible combinations. Surprisingly, no prototrophic colonies (putative hybrids) were formed between conspecific mutants. Their inability to form hybrids hinted at the possibility that the *M. andauensis* and *M. fructicola* type strains could be sexually isolated. To test the mating capabilities of the mutants, we tried to cross them with three auxotrophic mutants of the type strain of *M. pulcherrima*. All produced prototrophic colonies at the intersections with the *M. pulcherrima* mutants Mp5 and Mp13 but not with Mp12 (**Figure 8**). These results imply that the *M. andauensis* and *M. fructicola* mutants were not sterile but sexually incompatible with each other. When the *M. pulcherrima* mutants were mass-mated with each other, both Mp5 and Mp 13 produced prototrophs with Mp12, but not with each other (**Table 3**). Hence, the mutants of the three species could be divided in two groups of different mating activities. All *M. andauensis* and *M. fructicola* mutants as well as the *M. pulcherrima* mutant Mp12 formed one group and the *M. pulcherrima* mutants Mp5 and Mp13 formed a different group. Members of the same group could not hybridize (mate) with each other but could form hybrids with members of the other group (**Table 3**). This mating behavior is reminiscent of heterothallic mating types of other yeast species, designated “a” and “alpha” or “plus” and “minus.” We will therefore tentatively denote the larger group “a” and the smaller group “alpha.” Very rarely prototrophic colonies appeared at the intersections of strains belonging to the same mating type, but their frequency did not exceed the frequency of spontaneous revertants (**Figure 8A**). The sexual activities of ascomyceteous yeasts are determined by mating-type determinants referred to as *MATa* and *MATalpha* in most species. To identify the counterparts of these determinants we searched the *M. fructicola* genome sequence with the *Clavispora lusitaniae* orthologs (CLUG_04923, CLUG_02322 and CLUG_04271) of the *S. cerevisiae* proteins MATalpha1, MATalpha2, and HMRa1. The search detected ORFs coding for similar proteins. Thus, *M. fructicola* CBS 8853^T^ possesses counterparts of the central regulators of both mating types of *Saccharomyces*.

**Table 3:** Hybridisation of auxotrophic mutants and segregants.

Strains tested for hybridisation			Hybridisation partners (test strains)				
			
			*M. pulcherrima*			*M. andauensis*	*M. fructicola*
				
			Mp5 α	Mp12 a	Mp13 α	Ma22, 28, 32	Mf1456, 1458
			
			his^-^	ade^-^	lys^-^	ade^-^, arg^-^, try^-^	lys^-^ pro^-^, ade^-^ his^-^
*M. pulcherrima*	Mp5 α	his^-^	-^x^	+	-	+	+
	Mp12 a	ade^-^	+	-^x^	+	-	-
	Mp13 α	lys^-^	-	+	-^x^	+	+
*M. andauensis*	Ma22	ade^-^	+	-	+	-, -^x^	-
	Ma28	arg^-^	+	-	+	-, -^x^	-
	Ma32	try^-^	+	-	+	-, -^x^	-
*M. fructicola*	Mf1456	lys^-^ pro-	+	-	+	-	-, -^x^
	Mf1458	ade^-^ his^-^	+	-	+	-	-, -^x^
Ma22xMp5 segregants	S8	ade^-^	-	-^x^	-	+, -^x^	+
	S15	ade^-^	+	-^x^	+	-, -^x^	-
	S30	ade^-^	-	-^x^	-	+, -^x^	+
	S57	ade^-^ his^-^	-	-^x^	-	+, -^x^	+
Ma28xMp5 segregants	S2	his^-^	-^x^	+	-	+	+, -^x^
	S57	arg^-^	+	-	+	-, -^x^	-, -
	SQ	arg^-^ his^-^	-^x^	+	-	+, -^x^	+, -^x^


*+, prototrophs are produced at the intersections; x, match of identical auxotrophic markers.*

### Clamidospore Formation, Sporulation, and Segregation of Hybrids

**FIGURE 9:**
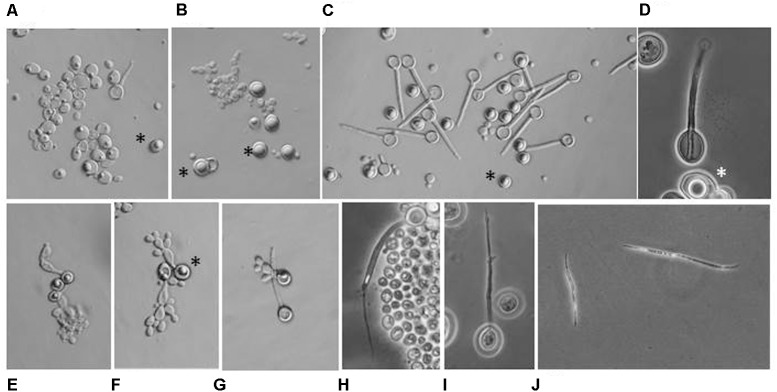
Microscopic morphology. **(A)** *M. andauensis* Ma22. Rudimentary asci with no discernable spores can occasionally seen among the cells. **(B)** *M. pulcherrima* Mp5. **(C)** Hybrid Ma22xMp5. **(D)** A two-spored ascus from the hybrid MA22xMp5. **(E)** Germinating pulcherrima cell (chlamydospore). **(F)** Pseudohypha formed by germinating pulcherrima cells. **(G)** Germination of pulcherrima cells is not accompanied with germination of ascospores. **(H)** Rare spontaneous release of ascospore. **(I)** Release of ascospore facilitated by Zymolyase treatment. **(J)** Free ascospores released by Zymolyase-treated asci. **(A–D)** Samples taken from cultures incubated on vegetable agar at 12°C for 4 weeks. **(E–H)** Sandwich cultures on YEA films incubated at 20°C. **(E–G)** After 72 h. **(H)** After 7 days. Stars denote pulcherrima cells (chlamydospores).

Both *M. andauensis* and *M. fructicola* were described as species producing asci containing needle-shape ascospores (Kurtzman and Droby, 2001; Molnar and Prillinger, 2005) and pulcherrima cells assumed to be the forms from which asci can develop (Pitt and Miller, 1968). We found that all mutant and hybrid cultures contained 5 to 70% pulcherrima cells when cultivated on vegetable agar at low temperature. We did not observe conjugation between pulcherrima cells or between budding cells. Conjugation was not observed in samples taken from intersections of the hybridisation grids either. The mutants formed asci with frequencies <0.1% but most asci contained no discernible spores (**Figures 9A,B**). The *M. andauensis* and *M. pulcherrima* hybrids formed up to 25% asci which were much larger than the asci of the parental mutants and usually had two spores (**Figures 9C,D**). Spontaneous ascus autolysis was observed very rarely (**Figure 9H**). Treatment with the cell-wall lytic enzyme Zymolyase degraded the ascus wall and resulted in free spores (**Figures 9I,J**). 80 individual intact asci and 70 Zymolyase-released spores were separated by micromanipulation from samples transferred on YEA plates and monitored for 4 weeks at room temperature. None of the spores germinated. No spore germination was observed in hundreds of asci and free spores released from the asci by Zymolyase treatment on YEA films sandwiched between glass slides and cover slips either. In contrast, the pulcherrima cells germinated in the sandwich cultures (**Figure 9E**) and formed pseudohyphae (**Figures 9F,G**) which then gradually returned to the standard budding morphology after a couple of cell divisions. DAPI-staining detected fluorescent material in most ascospores similar to that visible in the nuclei of vegetative cells. No germination was observed in samples frozen before spreading on YEA films either. We tested the effect of freezing because pigmented *Metschnikowia* strains are common mainly in climatic zones with harsh winter conditions.

Although the spores seemed dormant, we checked the sporulating cultures of the hybrids for segregation by spreading their cells on a complete medium to obtain individual single-cell colonies. After replica-plating the colonies on a minimal medium, we found 4 and 14 auxotrophic colonies among 950 colonies of the Ma22 × Mp5 and Ma28 × Mp5 hybrids, respectively (**Table 3**). In both cases one of the prototrophs was recombinant showing the auxotrophies of both parents. The segregants were then tested also for mating activity. All formed prototrophic hybrids with mutants of only one or the other mating type. Thus, they were heterothallic like their parental strains, but in some of them the parental marker and the parental mating type separated. For example, all segregants of the Ma22 × Mp5 hybrid were ade^-^ but only one showed the mating activity of the *M. andauensis* parental strain. No segregation was detected among 1000 colonies when the *M. fructicola* × *M. pulcherrima* hybrids were tested.

## Discussion

In a previous study we found that the type strains of *M. andauensis* and *M fructicola* had highly heterogeneous LSU (26S) rRNA gene paralogs that formed a continuous pool leaving no barcode gap suitable for differentiation of these two species by D1/D2 sequencing (Sipiczki et al., 2013). In this work we extended the investigations to the ITS1-5.8S-ITS2 regions of their rDNA repeats. We found that like the D1/D2 domains, both ITS spacers of both strains had ambiguous nucleotides when the regions were sequenced directly from the genomes. To resolve the observed sequence ambiguities, we randomly cloned individual DNA molecules from the amplified DNA preparation and determined the sequence of each clone. All *M. andauensis* clones and most *M. fructicola* clones had unique sequences. The revealed polymorphism cannot be attributed to sequencing errors because the variable sites were not scattered randomly across the cloned regions. When we blasted the cloned sequences against the draft genome sequence (Hershkovitz et al., 2013) of the *M. fructicola* type strain, we found additional sequence versions. Identity with a cloned sequence was found only in one genomic locus. Interestingly, certain *M. fructicola* genomic sequences were more similar to certain *M. andauensis* clones than to their own paralogs. This finding reinforces the notion based on D1/D2 analysis (Sipiczki et al., 2013) that these organisms share a pool of non-homogenized rDNA repeats.

Intragenomic (within-individual) ITS diversity has been detected in a small percentage of fungi. In a recent analysis by pyrosequencing of ITS amplicons of single-spore clones of 44 Ascomycota and 55 Basidiomycota species, Lindner et al. (2013) found significant intragenomic ITS polymorphism in only four species. The diversity in the *Metschnikowia* strains studied in this work was much bigger (14.7%) than the intragenomic diversity detected in the genome sequences of filamentous fungi and mushrooms for which data are available (e.g., O’Donnell and Cigelnik, 1997; Ko and Jung, 2002; Kauserud and Schumacher, 2003; Kauserud et al., 2007; Simon and Weiss, 2008; Kovacs et al., 2011; Lindner and Banik, 2011; Vydryakova et al., 2012; Lindner et al., 2013). Intragenomic ITS polymorphism of this extent has not been described in yeasts either, although ITS heterogeneity has been reported for several species. The only yeast species in which comparable polymorphism was revealed is *Geotrichum candidum* (Alper et al., 2011). The intragenomic diversity (26 variable positions) detected in two *G. candidum* strains was only slightly lower than the diversity (34 polymorphic sites) in our *Metschnikowia* strains. In the more closely related species *Pichia membranifaciens* only two types of ITS1 and two types of ITS2 sequences were detected (Wu et al., 2006). Large-scale analysis of rDNA repeats in strains of *Ashbya gossypii*, *Cryptococcus neoformans* and two *Saccharomyces* species (Ganley and Kobayashi, 2007) detected differences among rDNA units but the number of polymorphic positions was very low and, unlike the pattern we report here, the polymorphic sites were scattered randomly throughout the entire repeat units. Zhao et al. (2015) identified four and three polymorphic sites in the ITS sequences of *Candida glabrata* and *P. norvegensis* clinical isolates. In a *Xanthophyllomyces* strain two ITS sequences differing by 2 nucleotides were identified (Fell et al., 2007). The intragenomic diversity of the *Metschnikowia* sequences determined is this study was so big that some of them turned out to be more different from certain paralogs than from their orthologs in certain taxonomically more distant species. Taking together the intragenomic D1/D2 diversity described previously and the ITS diversity revealed in this study, it can be concluded that rDNA sequencing cannot be used for the taxonomic separation of *M. andauensis* and *M. fructicola* from each other and from related species. The high intragenomic diversity may further cause difficulties when yeast communities techniques (e.g., metagenomic analysis) are applied to the investigation of environmental samples and can easily result in misinterpretation of the sequence data. In view of the detected high intragenomic diversity, all strains previously assigned to either of these species (e.g., Piombo et al., 2018) should be taxonomically re-examined. In this context it is worth mentioning that the original taxonomic description of both species was based on rDNA sequences containing numerous ambiguous nucleotides (Kurtzman and Droby, 2001; Molnar and Prillinger, 2005).

The search of the *M. fructicola* genome sequence for ITS sequences identified 8 rDNA units in 6 sites. One of the sites contained a complete solo rDNA unit, whereas the rest of the sites looked like termini of arrays. As the proximal adjacent sequences of the units differed from each other, it is unlikely that the rDNA of this strain is organized in a single array. Its rDNA units seem to be dispersed in at least five distinct locations, but given the fact that the genome is not completely sequenced and assembled yet (Hershkovitz et al., 2013), the actual number of loci containing rDNA genes may be even higher. In other yeast species examined in detail, the rDNA units are organized in large continuous rDNA arrays and the intragenomic polymorphism is very low due to a process referred to as homogenisation or concerted evolution. In concerted evolution, the rRNA units evolve “horizontally,” meaning that a non-harmful mutation that arises in one unit spreads to all other units of the array. As a result, the units of the rDNA array do not evolve independently. The poorly explored underlying mechanism is assumed to involve unequal homologous recombination by sister-chromosome exchange, interchromosomal exchange, intrachromosomal exchange/deletion between rDNA units of the array(s) (for a review, see Eickbush and Eickbush, 2007) supported by cohesin (to hold broken sister-chromatid ends in place) and the condensation of the rDNA (to ensure proper segregation of the region in the M phase of the cell cycle) (for a review, see Kobayashi, 2006). I has been shown by computer modeling that homogenisation reduces the mutational load (Ohta, 1989). In *M. fructicola* the rDNA seems to have fragmented organization which may preclude the homogenisation of the physically separated shorter tandems and orphaned copies. This assumption is consistent with the results of a recent study of four *Nikotiana* species which found correlation between the number of ITS1 ribotypes and the number of locations of rDNA units in the genome (Matyasek et al., 2012). It was proposed that homogenisation can be higher within the arrays than between arrays and the low levels of homogenisation between different chromosomal loci would allow the accumulation of different ribotypes.

Given that the transcripts of both spacers are known to form structures required for correct processing of the transcripts of the rRNA genes (Hillis and Dixon, 1991; Nazar, 2004; Mullineux and Hausner, 2009), the question arises whether all paralogs found in the *Metschnikowia* strains code for functional ITS transcripts. To answer this question we examined the diversity of the ITS1 and ITS2 secondary structures of all clones and genomic sequences. We found that substitutions at the polymorphic sites of ITS2 regions had no effect on the predicted secondary structure. This structural conservation is consistent with the notion that ITS2 is more strictly conserved than ITS1 in the majority of fungi (e.g., Beiggi and Piercey-Normore, 2007; Nilsson et al., 2008) because it is under stronger selective constraints. Typically, this spacer exhibits a low level of sequence variation within species and a high level of divergence between species (for a review see Coleman, 2015). This feature renders the ITS2 sequences useful for taxonomic differentiation and inference of phylogenetic relationships of different taxa. However, in our case the *M. andauensis* and *M. fructicola* ITS2 sequences did not form distinct groups. The ITS1 segments had much more polymorphic sites, and hence the predicted secondary structures of their transcripts showed high level of diversity. It can be assumed that the ITS1-5.8S-ITS2 sequence found in 5 *M. fructicola* clones and one genomic locus might be the “functional standard” in *M. fructicola*. It was specified as ancestral in the parsimony network analysis and its accompanying D1/D2 sequence was previously found among the cloned D1/D2 domains (Sipiczki et al., 2013). This and the versions differing from it only by a few substitutions with only minor effects on the ITS1 secondary structure may supply the cell with a functional pool of transcripts. Although the positions of the variable sites showed some tendency to accumulate in certain segments, pairs of compensatory substitutions (coevolving pairs of sites) could not be identified. This is a fundamental difference from the D1/D2 domains, in which coevolving pairs of nucleotides in the pairing stretches of loops appeared to safeguard structural stability (Sipiczki et al., 2013). Since the RNA self-splicing activity of ITS1 is also important for the proper assembly of ribosomes (through its role in the maturation of the 18S rRNA) (van Nues et al., 1994), the rDNA units encoding ITS1 transcripts of strongly divergent secondary structures might negatively interfere with the “functional standard” in ribosome genesis. This effect can be avoided by silencing and inactivation of these units. Attenuation and silencing preclude or at least hampers the production of aberrant transcripts and thus can protect the cell against the adverse effect of abnormally spliced rRNAs on ribosome formation. At the same time, the activity reduction relieves these rDNA units from the selection pressure, allowing the fixation of all types of mutations with equal probabilities in any part of their sequences regardless of the structural consequences. Accumulation of mutations can gradually degenerate the inactivated rDNA units into non-functional pseudogenes. Pseudogenes are genomic sequences that arise from functional genes but their genetic defects (e.g., mutations) preclude the generation of functional transcripts (for a review, see Balakirev and Ayala, 2003). In plants, non-functional rDNA units containing pseudogenes are recognizable by their irregular 5.8S sequences and the absence of some of the ITS regions (Queiroz et al., 2011). We found this kind of truncate structures in two unitigs of the *M. fructicola* genome sequence; both lacked ITS1 and the nucleotide sequences of their 5.8S genes were very different from those of the other rDNA units. We assume that these rDNA units may be remnants of erstwhile active units being in an advanced stage of decay progressing in a way analogous to that observed e.g., in *Buchnera* (Degnan et al., 2011). rDNA pseudogenes occur in high numbers in certain plants (e.g., Bailey et al., 2003; Harpke and Peterson, 2007) but were detected only in few filamentous fungi (e.g., Rooney and Ward, 2005; Lindner and Banik, 2011; Lindner et al., 2013; Li et al., 2017), all being unrelated to *Metschnikowia*. The dispersed locations of rDNA units in the genome and the presence of truncated copies (pseudogenisation) indicate that the sequence homogeneity of the ITS1-5.8S-ITS2 region of *M. fructicola* is not maintained by homogenisation, and that its rDNA units evolve under a birth-and-death process. In the latter mode of evolution it is selection, not homogenisation that maintains the rDNA units as a coherent family. New copies of genes are created by gene duplications and some duplicated genes are preserved in the genome for long periods of time, whereas others become non-functional through the accumulation of harmful mutations and undergo gradual degradation (for a review, see Nei and Rooney, 2005). The high divergence among the sequences cloned from the *M. andauensis* strain implies that its rDNA is also shaped by birth-and-death evolution. Nevertheless, the rDNA units corresponding to cloned sequences with divergent secondary structures can only be in early phases of pseudogenisation because they still have complete ITS segments and their 5.8S genes differ from each other only at one site. To our knowledge this is the first report of birth-end-death evolution of rDNA in yeasts.

The results of the phylogenetic analysis of the D1/D2 domains of these two species suggested that their non-homogenized 26S rRNA genes had not evolved in a vertical tree-like way but most probably by reticulate intra- and interspecies interactions (Sipiczki et al., 2013). The findings of this study demonstrate that the ITS regions of the repeats are also involved in the reticulation. Neither the statistical parsimony network analysis nor the phylogenetic network analyses grouped the sequences of the strains in distinct clades. Their intermixing in both types of networks indicated that the rDNA units of these strains must have evolved in interaction. For genetic interactions the units of the strains need to be brought together and then separated. As horizontal gene transfer by mechanisms widespread in bacteria is unlikely to take place in *Metschnikowia*, hybridisation can be assumed to be the major mechanism of bringing the two sets of rDNA units together. Both strains form ascospores (Kurtzman and Droby, 2001; Molnar and Prillinger, 2005) suggesting that both possess sexual phases, in which they can hybridize, provided they are sexually compatible.

In an attempt to test them for sexual compatibility, we generated mutants with complementary auxotrophies from their cultures and co-cultivated them in all possible pair-wise combinations. Unexpectedly, no prototrophs were produced in any combinations as if the mutants were sterile. To find out whether the failure was due to sterility, we crossed them also with three mutants of the related species *M. pulcherrima*. All *M. andauensis* and *M. fructicola* mutants formed prototrophs with two *M. pulcherrima* mutants but not with the third mutant. Hence, neither the *M. andauensis* nor the *M. fructicola* mutants were sterile. The two *M. pulcherrima* mutants compatible with them did not form hybrids with each other but did with the third mutant. These finding implies that all mutants of the three species were heterothallic, and that all *M. andauensis* and *M. fructicola* mutants as well as the *M. pulcherrima* mutant not hybridizing with them had identical mating type. The inability of their mutants to hybridize with each other, however, does not necessarily mean that *M. andauensis* and *M. fructicola* are sexually isolated. Both produce asci in pure cultures, albeit at low frequencies, and preliminary results suggested that *M. fructicola* be diploid and heterozygous at the mating type locus (Lachance, 2016). We found potential orthologs of both *MATa* and *MATalpha* ORFs in its genome sequence, but their simultaneous presence does not prove that the strain is heterozygous diploid. In numerous other yeast species, heterothallic haploids can have both mating-type determinants, but usually only one is active (e.g., Haber, 2012; Watanabe et al., 2013). Thus, we most probably had heterothallic haploid mutants of identical mating types, indicating that the type strains analyzed may not be homothallic and their cultures may also contain mating-proficient haploid cells. Interestingly, all mutants of the three species formed “pulcherrima cells” (chlamydospores). It was previously proposed that diploid cells develop into chlamydospores (Lachance, 2016). Their abundance in the mutant cultures implies that either haploids can also convert to chlamydospores or the heterothallic haploid cells frequently double their genomes by autodiploidisation. The low sporulation efficiency in the wild-type cultures also argues for the predominance of haploidy because the hybrids with the *M. pulcherrima* mutants showed very high sporulation activity. Somewhat unexpectedly, the spores of the hybrids did not germinate. This can be attributed to a postzygotic sterility barrier analogous to that operating in the allodiploid hybrids of *Saccharomyces* species isolated by a double sterility barrier (e.g., Pfliegler et al., 2012). Alternatively, the failure of the ascospores to germinate might be due endogenous inhibitors keeping the spores “dormant.” Certain fungi were found to produce “endogenously dormant spores” whose dormancy can be broken by aging or by some physiological shock permitting the endogenous inhibitors to leach out or degrade (e.g., Graham et al., 2014). We tested only one potential shock. Freezing and thawing did not activate the spores.

Nevertheless, the isolation is not absolute because segregants with parental markers and marker combinations were found at low frequencies in the sporulating cultures of the hybrids. It is not clear whether these segregants were clones produced by a few germinating spores or products of mitotic segregation. The latter possibility would be consistent with the results of a recent reinterpretation of some early observations that suggest that *M. pulcherrima* may have a parasexual life cycle (Naumov, 2011). In the model proposed by Naumov (2011), both mitotic (parasexual) and meiotic (sexual) haploidisation can take place in the life cycle of *Metschnikowia* sensu stricto. Although only indirectly, through the involvement of a third species, the hybridisation experiments prove that the type strains of *M. andauensis* and *M. fructicola* are not completely isolated and hence interspecies (intergenomic) reticulation-type interactions can be involved in the evolution of their rDNA genes. It is worth mentioning here that James et al. (2009) noted that in *Saccharomyces* many single nucleotide polymorphisms (SNPs) were more common in strains with mosaic/hybrid genomes than in strains with standard genomes, suggesting that hybridization plays a role in the evolution of intragenomic variation even in yeasts having large rDNA arrays suitable for homogenisation.

## Author Contributions

MS conceived and designed the experiments, performed the bioinformatics analysis, and wrote the manuscript. MS, EH, and WP performed the experiments. All authors reviewed the manuscript.

## Conflict of Interest Statement

The authors declare that the research was conducted in the absence of any commercial or financial relationships that could be construed as a potential conflict of interest.

## Footnotes

1 http://blast.ncbi.nlm.nih.gov/Blast.cgi

2 https://unite.ut.ee/analysis.php

3 https://www.ncbi.nlm.nih.gov/genome/

4 http://unafold.rna.albany.edu/?q=mfold

5 http://its2.bioapps.biozentrum.uni-wuerzburg.de/

6 https://www.yeastgenome.org/search?q=ITS1%20ITS2&is_quick=true

## References

[B1] Alper I. Frenette M. Labrie S. (2011). Ribosomal DNA polymorphisms in the yeast *Geotrichum candidum*. *Fungal Biol.* 115 1259–1269. 10.1016/j.funbio.2011.09.002 22115445

[B2] Ankenbrand M. J. Keller A. Wolf M. Schultz J. Förster F. (2015). ITS2 Database V: twice as much. *Mol. Biol. Evol.* 32 3030–3032. 10.1093/molbev/msv174 26248563

[B3] Bailey C. D. Carr T. G. Harris S. A. Hughes C. E. (2003). Characterization of angiosperm nrDNA polymorphism, paralogy, and pseudogenes. *Mol. Phylogenet. Evol.* 29 435–455. 10.1016/j.ympev.2003.08.021 14615185

[B4] Balakirev E. S. Ayala F. J. (2003). Pseudogenes: are they ‘junk’ or functional DNA? *Annu. Rev. Genet.* 37 123–151. 10.1146/annurev.genet.37.040103.103949 14616058

[B5] Beiggi S. Piercey-Normore M. D. (2007). Evolution of ITS ribosomal RNA secondary structures in fungal and algal symbionts of selected species of *Cladonia* sect. *Cladonia* (Cladoniaceae, Ascomycotina). *J. Mol. Evol.* 64 528–542. 10.1007/s00239-006-0115-x 17460809

[B6] Bruen T. Philippe H. Bryant D. (2006). A quick and robust statistical test to detect the presence of recombination. *Genetics* 172 2665–2681. 10.1534/genetics.105.048975 16489234 PMC1456386

[B7] Bryant D. Moulton V. (2004). Neighbor-Net: an agglomerative method for the construction of phylogenetic networks. *Mol. Biol. Evol.* 21 255–265. 10.1093/molbev/msh018 14660700

[B8] Caetano-Anolle’s G. (2002). Tracing the evolution of RNA structures in ribosomes. *Nucleic Acids Res.* 30 2575–2587. 10.1093/nar/30.11.2575 12034847 PMC117177

[B9] Clement M. Posada D. Crandall K. A. (2000). TCS: a computer program to estimate gene genealogies. *Mol. Ecol.* 9 1657–1660. 10.1046/j.1365-294x.2000.01020.x 11050560

[B10] Coleman A. W. (2015). Nuclear rRNA transcript processing versus internal transcribed spacer secondary structure. *Trends Genet.* 31 157–163. 10.1016/j.tig.2015.01.002 25648500

[B11] Cook A. H. Slater C. A. (1956). The structure of pulcherrimin. *J. Chem. Soc.* 4133–4135. 10.1039/jr9560004133

[B12] Cryle M. J. Bell S. G. Schlichting I. (2010). Structural and biochemical characterization of the cytochrome P450 CypX (CYP134A1) from *Bacillus subtilis*: a cyclo-L-leucyl-L-leucyl dipeptide oxidase. *Biochemistry* 49 k7282–7296. 10.1021/bi100910y 20690619

[B13] Degnan P. H. Ochman H. Moran N. A. (2011). Sequence conservation and functional constraint on intergenic spacers in reduced genomes of the obligate symbiont *Buchnera*. *PLoS Genet.* 7:e1002252. 10.1371/journal.pgen.1002252 21912528 PMC3164680

[B14] Eickbush T. H. Eickbush D. G. (2007). Finely orchestrated movements: evolution of the ribosomal RNA genes. *Genetics* 175 477–485. 10.1534/genetics.107.071399 17322354 PMC1800602

[B15] Elder J. F. Turner B. J. (1995). Concerted evolution of repetitive DNA sequences in eukaryotes. *Q. Rev. Biol.* 70 297–320. 10.1086/419073 7568673

[B16] Fell J. W. Scorzetti G. Statzell-Tallman A. Boundy-Mills K. (2007). Molecular diversity and intragenomic variability in the yeast genus *Xanthophyllomyces*: the origin of *Phaffia rhodozyma*? *FEMS Yeast Res.* 7 1399–1408. 17825066 10.1111/j.1567-1364.2007.00297.x

[B17] Ganley A. R. Kobayashi T. (2007). Highly efficient concerted evolution in the ribosomal DNA repeats: total rDNA repeat variation revealed by whole-genome shotgun sequence data. *Genome Res.* 17 184–191. 10.1101/gr.5457707 17200233 PMC1781350

[B18] Gorodkin J. Heyer L. J. Brunak S. Stormo G. D. (1997). Displaying the information contents of structural RNA alignments: the structure logos. *Comput. Appl. Biosci.* 13 583–586. 9475985 10.1093/bioinformatics/13.6.583

[B19] Graham J. K. Smith M. L. Simons A. M. (2014). Experimental evolution of bet hedging under manipulated environmental uncertainty in *Neurospora crassa*. *Proc. R. Soc. B Biol. Sci.* 281:20140706. 10.1098/rspb.2014.0706 24870047 PMC4071552

[B20] Granneman S. Petfalski E. Tollervey D. (2011). A cluster of ribosome synthesis factors regulate pre-rRNA folding and 5.8S rRNA maturation by the Rat1 exonuclease. *EMBO J.* 19 4006–4019. 10.1038/emboj.2011.256 21811236 PMC3209772

[B21] Guzmán B. Lachance M. A. Herrera C. M. (2013). Phylogenetic analysis of the angiosperm-floricolous insect-yeast association: have yeast and angiosperm lineages co-diversified? *Mol. Phylogenet. Evol.* 68 161–175. 10.1016/j.ympev.2013.04.003 23583418

[B22] Haber J. E. (2012). Mating-type genes and *MAT* switching in *Saccharomyces cerevisiae*. *Genetics* 191 33–64. 10.1534/genetics.111.134577 22555442 PMC3338269

[B23] Harpke D. Peterson A. (2007). Quantitative PCR revealed a minority of ITS copies to be functional in *Mammillaria* (Cactaceae). *Int. J. Plant Sci.* 168 1157–1160. 10.1086/520729

[B24] Hasegawa M. Kishino H. Yano T. (1985). Dating of human-ape splitting by a molecular clock of mitochondrial DNA. *J. Mol. Evol.* 22 160–174. 10.1007/BF02101694 3934395

[B25] Hershkovitz V. Sela N. Taha-Salaime L. Liu J. Rafael G. Kessler C. (2013). De-novo assembly and characterization of the transcriptome of *Metschnikowia fructicola* reveals differences in gene expression following interaction with *Penicillium digitatum* and grapefruit peel. *BMC Genomics* 14:168. 10.1186/1471-2164-14-168 23496978 PMC3608080

[B26] Hillis D. M. Dixon M. T. (1991). Ribosomal DNA: molecular evolution and phylogenetic inference. *Q. Rev. Biol.* 66 411–453. 10.1086/417338 1784710

[B27] James S. A. O’Kelly M. J. T. Carter D. M. Davey R. P. van Oudenaarden A. Roberts I. N. (2009). Repetitive sequence variation and dynamics in the ribosomal DNA array of *Saccharomyces cerevisiae* as revealed by whole-genome resequencing. *Genome Res.* 19 626–635. 10.1101/gr.084517.108 19141593 PMC2665781

[B28] Kauserud H. Schumacher T. (2003). Ribosomal DNA variation, recombination and inheritance in the basidiomycete *Trichaptum abietinum*: implications for reticulate evolution. *Heredity* 91 163–172. 10.1038/sj.hdy.6800294 12886283

[B29] Kauserud H. Vegården I. B. Decock C. Hallenberg N. (2007). Hybridization among cryptic species of the cellar fungus *Coniophora puteana* (Basidiomycota). *Mol. Ecol.* 16 389–399. 10.1111/j.1365-294X.2006.03129.x 17217352

[B30] Kerpedjiev P. Hammer S. Hofacker I. L. (2015). Forna (force-directed RNA): simple and effective online RNA secondary structure diagrams. *Bioinformatics* 31 3377–3379. 10.1093/bioinformatics/btv372 26099263 PMC4595900

[B31] Kluyver A. J. van der Walt J. P. van Triet A. J. (1953). Pulcherrimin, the pigment of *Candida pulcherrima*. *Proc. Natl. Acad. Sci. U.S.A.* 39 583–593. 10.1073/pnas.39.7.583 16589308 PMC1063828

[B32] Ko K. S. Jung H. S. (2002). Three nonorthologous ITS1 types are present in a polypore fungus *Trichaptum abietinum*. *Mol. Phylogenet. Evol.* 23 112–122. 10.1016/S1055-7903(02)00009-X 12069544

[B33] Kobayashi T. (2006). Strategies to maintain the stability of the ribosomal RNA gene repeats–collaboration of recombination, cohesion, and condensation. *Genes Genet. Syst.* 81 155–161. 10.1266/ggs.81.155 16905869

[B34] Kovacs G. M. Balazs T. K. Calonge F. D. Martín M. P. (2011). The diversity of *Terfezia* desert truffles: new species and a highly variable species complex with intrasporocarpic nrDNA ITS heterogeneity. *Mycologia* 103 841–853. 10.3852/10-312 21289106

[B35] Kurtzman C. P. Droby S. (2001). *Metschnikowia fructicola*, a new ascosporic yeast with potential for biocontrol of postharvest fruit rots. *Syst. Appl. Microbiol.* 24 395–399. 10.1078/0723-2020-00045 11822675

[B36] Kurtzman C. P. Robnett C. J. Basehoar E. (2018). Four new species of the yeast genus *Metschnikowia* and the transfer of seven *Candida* species to *Metschnikowia* and *Clavispora* as new combinations. *Antonie Van Leeuwenhoek* [Epub ahead of print] 10.1007/s10482-018-1095-8 29754318

[B37] Lachance M. A. (2011). “*Metschnikowia* Kamienski (1899),” in *The Yeasts. A Taxonomic Study*, eds Kurtzman C. P. Fell J. W. Boekhout T. (Amsterdam: Elsevier), 575–620.

[B38] Lachance M. A. (2016). *Metschnikowia*: half tetrads, a regicide and the fountain of youth. *Yeast* 33 563–574. 10.1002/yea.3208 27599462

[B39] Li Y. Yang R. H. Jiang L. Hu X. D. Wu Z. J. Yao Y. J. (2017). rRNA Pseudogenes in filamentous ascomycetes as revealed by genome data. *G3* 7 2695–2703. 10.1534/g3.117.044016 28637809 PMC5555474

[B40] Lindner D. L. Banik M. T. (2011). Intragenomic variation in the ITS rDNA region obscures phylogenetic relationships and inflates estimates of operational taxonomic units in genus *Laetiporus*. *Mycologia* 103 731–740. 10.3852/10-331 21289107

[B41] Lindner D. L. Carlsen T. Nilsson R. H. Davey M. Schumacher T. Kauserud H. (2013). Employing 454 amplicon pyrosequencing to reveal intragenomic divergence in the internal transcribed spacer rDNA region in fungi. *Ecol. Evol.* 3 1751–1764. 10.1002/ece3.586 23789083 PMC3686207

[B42] Markham N. R. Zuker M. (2008). UNAFold: software for nucleic acid folding and hybridization. *Methods Mol. Biol.* 453 3–31. 10.1007/978-1-60327-429-6_1 18712296

[B43] Matyasek R. Renny-Byfield S. Fulnecek J. Macas J. Grandbastien M. A. Nichols R. (2012). Next generation sequencing analysis reveals a relationship between rDNA unit diversity and locus number in *Nicotiana diploids*. *BMC Genomics* 13:722. 10.1186/1471-2164-13-722 23259460 PMC3563450

[B44] Molnar O. Prillinger H. (2005). Analysis of yeast isolates related to *Metschnikowia pulcherrima* using the partial sequence of the large subunit rDNA and the actin gene; description of *Metschnikowia andauensi* sp. *Syst. Appl. Microbiol.* 28 717–726. 10.1016/j.syapm.2005.05.009 16261861

[B45] Mullineux T. Hausner G. (2009). Evolution of rDNA ITS1 and ITS2 sequences and RNA secondary structures within members of the fungal genera *Grosmannia* and *Leptographium*. *Fungal Genet. Biol.* 46 855–867. 10.1016/j.fgb.2009.08.001 19665572

[B46] Naumov G. I. (2011). Molecular and genetic differentiation of small-spored species of the yeast genus *Metschnikowia* Kamienski. *Mikrobiologiya* 80 147–154. 10.1134/S0026261711020111 21774178

[B47] Nazar R. N. (2004). Ribosomal RNA processing and ribosome biogenesis in eukaryotes. *IUBMB Life* 56 457–465. 10.1080/15216540400010867 15545225

[B48] Nei M. Rooney A. P. (2005). Concerted and birth-and-death evolution of multigene families. *Annu. Rev. Genet.* 39 121–152. 10.1146/annurev.genet.39.073003.112240 16285855 PMC1464479

[B49] Nilsson R. H. Kristiansson E. Ryberg M. Hallenberg N. Larsson K.-H. (2008). Intraspecific *ITS* variability in the kingdom Fungi as expressed in the international sequence databases and its implications for molecular species identification. *Evol. Bioinformatics* 4 193–201. 10.4137/EBO.S653 19204817 PMC2614188

[B50] O’Donnell K. Cigelnik E. (1997). Two divergent intragenomic rDNA ITS2 types within a monophyletic lineage of the fungus *Fusarium* are nonorthologous. *Mol. Phylogenet. Evol.* 7 103–116. 10.1006/mpev.1996.0376 9007025

[B51] Ohta T. (1989). The mutational load of a multigene family with uniform members. *Genet. Res.* 53 141–145. 10.1017/S0016672300028020 2744453

[B52] Pfliegler W. P. Antunovics Z. Sipiczki M. (2012). Double sterility barrier between *Saccharomyces* species and its breakdown in allopolyploid hybrids by chromosome loss. *FEMS Yeast Res.* 12 703–718. 10.1111/j.1567-1364.2012.00820.x 22697168

[B53] Piombo E. Sela N. Wisniewski M. Hoffmann M. Gullino M. L. Allard M. W. (2018). Genome sequence, assembly and characterization of two *Metschnikowia fructicola* strains used as biocontrol agents of postharvest diseases. *Front. Microbiol.* 9:593. 10.3389/fmicb.2018.00593 29666611 PMC5891927

[B54] Pitt J. I. Miller M. W. (1968). Sporulation in *Candida pulcherrima*, *Candida reukaufii* and *Chlamydozyma* species: their relationships with *Metschnikowia*. *Mycologia* 60 663–685. 10.2307/3757434

[B55] Queiroz C. S. Batista F. R. C. Oliveira L. O. (2011). Evolution of the 5.8S nrDNA gene and internal transcribed spacers in *Carapichea ipecacuanha* (Rubiaceae) within a phylogeographic context. *Mol. Phylogenet. Evol.* 59 293–302. 10.1016/j.ympev.2011.01.013 21300163

[B56] Rooney A. P. Ward T. J. (2005). Evolution of a large ribosomal RNA multigene family in filamentous fungi: birth and death of a concerted evolution paradigm. *Proc. Natl. Acad. Sci. U.S.A.* 102 5084–5089. 10.1073/pnas.0409689102 15784739 PMC555991

[B57] Schlotterer C. Hauser M.-T. von Haeseler A. Tautz D. (1994). Comparative evolutionary analysis of rDNA ITS regions in *Drosophila*. *Mol. Biol. Evol.* 11 513–522. 8015444 10.1093/oxfordjournals.molbev.a040131

[B58] Schoch C. L. Seifert K. A. Huhndorf A. Robert V. Spouge J. L. Levesque C. A. (2012). Nuclear ribosomal internal transcribed spacer (ITS) region as a universal DNA barcode marker for Fungi. *Proc. Natl. Acad. Sci. U.S.A.* 109 6241–6246. 10.1073/pnas.1117018109 22454494 PMC3341068

[B59] Schultz J. Maisel S. Gerlach D. Müller T. Wolf M. (2005). A common core of secondary structure of the internal transcribed spacer 2 (ITS2) throughout the Eukaryota. *RNA* 11 361–364. 10.1261/rna.7204505 15769870 PMC1370725

[B60] Simon U. K. Weiss M. (2008). Intragenomic variation of fungal ribosomal genes is higher than previously thought. *Mol. Biol. Evol.* 25 2251–2254. 10.1093/molbev/msn188 18728073

[B61] Sipiczki M. (2003). *Candida zemplinina* sp. nov., an osmotolerant and psychrotolerant yeast that ferments sweet botrytized wines. *Int. J. Syst. Evol. Microbiol.* 53 2079–2083. 10.1099/ijs.0.02649-0 14657149

[B62] Sipiczki M. (2006). *Metschnikowia* strains isolated from botrytized grapes antagonize fungal and bacterial growth by iron depletion. *Appl. Environ. Microbiol.* 72 6716–6724. 10.1128/AEM.01275-06 17021223 PMC1610289

[B63] Sipiczki M. (2012). *Pichia bruneiensis* sp. nov., a biofilm-producing dimorphic yeast species isolated from flowers in Borneo. *Int. J. Syst. Evol. Microbiol.* 62 3099–3104. 10.1099/ijs.0.044974-0 22888193

[B64] Sipiczki M. Ferenczy L. (1978). Enzymic methods for enrichment of fungal mutants I. Enrichment of *Schizosaccharomyces pombe* mutants. *Mutat. Res.* 50 163–173. 10.1016/0027-5107(78)90021-0

[B65] Sipiczki M. Pfliegler W. P. Holb I. J. (2013). *Metschnikowia* species share a pool of diverse rRNA genes differing in regions that determine hairpin-loop structures and evolve by reticulation. *PLoS One* 8:e67384. 10.1371/journal.pone.0067384 23805311 PMC3689696

[B66] Sipiczki M. Takeo K. Yamaguchi M. Yoshida S. Miklos I. (1998). Environmentally controlled dimorphic cycle in a fission yeast. *Microbiology* 144 1319–1330. 10.1099/00221287-144-5-1319 9611807

[B67] Thompson J. D. Higgions D. G. Gibson T. J. (1994). CLUSTALW: improving the sensitivity of progressive multiple sequence alignment through sequence weighting, position-specific gap penalties and weight matrix choice. *Nucleic Acids Res.* 22 4673–4680. 10.1093/nar/22.22.4673 7984417 PMC308517

[B68] Torres-Machorro A. L. Hernandez R. Cevallos A. M. Lopez-Villasenor I. (2010). Ribosomal RNA genes in eukaryotic microorganisms: witnesses of phylogeny? *FEMS Microbiol. Rev.* 34 59–86. 10.1111/j.1574-6976.2009.00196.x 19930463

[B69] van Nues R. W. Rientjes J. M. J. van der Sande C. A. F. M. Zerp S. F. Sluiter C. Venema J. (1994). Separate structural elements within internal transcribed spacer 1 of *Saccharomyces cerevisiae* precursor ribosomal RNA direct the formation of 17S and 26S rRNA. *Nucleic Acids Res.* 22 912–919. 10.1093/nar/22.6.912 8152921 PMC307909

[B70] Venema J. Tollervey D. (1999). Ribosome synthesis in *Saccharomyces cerevisiae*. *Annu. Rev. Genet.* 33 261–311. 10.1146/annurev.genet.33.1.261 10690410

[B71] Vydryakova G. A. Van D. T. Shoukouhi P. Psurtseva N. V. Bissett J. (2012). Intergenomic and intragenomic ITS sequence heterogeneity in *Neonothopanus nambi* (Agaricales) from Vietnam. *Mycology* 3 89–99.

[B72] Watanabe J. Uehara K. Mogi Y. (2013). Diversity of mating-type chromosome structures in the yeast *Zygosaccharomyces rouxii* caused by ectopic exchanges between *MAT*-like loci. *PLoS One* 8:e62121. 10.1371/journal.pone.0062121 23614024 PMC3628578

[B73] White T. J. Bruns T. Lee S. Taylor J. (1990). “Amplification and sequencing of fungal ribosomal RNA genes for phylogenetics,” in *PCR Protocols. A Guide to Methods and Applications*, eds Innis M. A. Gelfand D. H. Sninsky J. J. White T. J. (San Diego, CA: Academic Press), 315–322.

[B74] Wolf M. Achtziger M. Schultz J. Dandekar T. Müller T. (2005). Homology modeling revealed more than 20,000 rRNA internal transcribed spacer 2 (ITS2) secondary structures. *RNA* 11 1616–1623. 10.1261/rna.2144205 16244129 PMC1370847

[B75] Wu Z. W. Robert V. Bai F. Y. (2006). Genetic diversity of the *Pichia membranifaciens* strains revealed from rRNA gene sequencing and electrophoretic karyotyping, and the proposal of *Candida californica* comb. nov. *FEMS Yeast Res.* 6 305–311. 10.1111/j.1567-1364.2006.00029.x 16487352

[B76] Xue M. L. Zhang L. Q. Wang Q. M. Zhang J. S. Bai F. Y. (2006). *Metschnikowia sinensis* sp. nov., *Metschnikowia zizyphicola* sp. nov. and *Metschnikowia shanxiensis* sp. nov., novel yeast species from jujube fruit. *Int. J. Syst. Evol. Microbiol.* 56 2245–2250. 10.1099/ijs.0.64391-0 16957129

[B77] Zhao Y. Tsang C. C. Xiao M. Cheng J. Xu Y. Lau S. K. (2015). Intra-genomic internal transcribed spacer region sequence heterogeneity and molecular diagnosis in clinical microbiology. *Int. J. Mol. Sci.* 16 25067–25079. 10.3390/ijms161025067 26506340 PMC4632790

[B78] Zimmer E. A. Martin S. L. Beverley S. M. Kan Y. W. Wilson A. C. (1980). Rapid duplication and loss of genes coding for the chains of hemoglobin. *Proc. Natl. Acad. Sci. U.S.A.* 77 2158–2162. 10.1073/pnas.77.4.2158 6929543 PMC348671

[B79] Zuker M. (2003). Mfold web server for nucleic acid folding and hybridization prediction. *Nucleic Acids Res.* 31 3406–3415. 10.1093/nar/gkg595 12824337 PMC169194

