# Supplementary material for: Birth-and-Death Evolution and Reticulation of ITS Segments of Metschnikowia andauensis and Metschnikowia fructicola rDNA Repeats
Source: Front Microbiol. 2018 Jun 12;9:1193. doi: 10.3389/fmicb.2018.01193 (PMC6005844; doi:10.3389/fmicb.2018.01193)
Supplement: Supplementary file 1 [file Table_1.DOCX]

Supplementary Material

**Birth-and-death Evolution and Reticulation of ITS Segments of *Metschnikowia andauensis* and *Metschnikowia* *fructicola* rDNA Repeats**

M. Sipiczki*, Eniko Horvath, W. P. Pfiegler

Department of Genetics and Applied Microbiology, University of Debrecen, Debrecen, Hungary

**Correspondence:**

M. Sipiczki

[gecela@post.sk](mailto:gecela@post.sk)

**Supplementary Table 1 ǀ List of strains**

| **Strain** | | **Genotype/**  **phenotype ^a^** | **Source** |
| --- | --- | --- | --- |
| *M. andauensis* | | | |
|  | 11-1120 (CBS 10809^T^) | Wild type | CBS |
|  | Ma22 | ade^-^ | This study |
|  | Ma23 | ade^-^ | This study |
|  | Ma28 | arg^-^ | This study |
|  | Ma32 | try^-^ | This study |
| *M. fructicola* | | | |
|  | 11-579 (CBS 8853^T^) | Wild type | CBS |
|  | Mf3 | pro^-^ | This study |
|  | Mf52 | his^-^ | This study |
|  | Mf1456 | lys^-^ pro^-^ | This study |
|  | Mf1458 | ade^-^  his^-^ | This study |
| *M. pulcherrima* | | | |
|  | 11-578 (CBS 5833^T^) | Wild type 578)rrimasections ed and inoculated onto fresh SMA plates to obtain pure hybrid cultureses was described | CBS |
|  | Mp5 | his^-^ | This study |
|  | Mp12 | ade^-^ | This study |
|  | Mp13 | lys^-^ | This study |
| *M. andauensis x M. pulcherrima* | | | |
|  | Ma22xMp5 hybrid | prototrophic | This study |
|  | Ma22xMp5-S8, S15, S30 segregants | ade^-^ | This study |
|  | Ma22xMp5-S54 segregant | ade^-^ his^-^ | This study |
|  | Ma28xMp5 hybrid | prototrophic | This study |
|  | Ma28xMp5-S2, 4a segregants | his^-^ | This study |
|  | Ma28xMp5-S57, 3b, 3c, 3d, 3e, 3g, 3h, 3i, 4b, 4c, 4d, 4g segregants | arg^-^ | This study |
|  | Ma28xMp5-SQ segregant | arg^-^ his^-^ | This study |
| *M. fructicola x M. pulcherrima* | | | |
|  | Mf1456xMp5 hybrid | prototrophic | This study |
|  | Mf1458xMp5 hybrid | prototrophic | This study |
| *M. pulchrrima x M. pulcherrima* | | | |
|  | Mp5xMp12 hybrid | prototrophic | This study |
|  | Mp12xMp13 hybrid | prototrophic | This study |

^T^ Type strain

CBS (Centraalbureau voor Schimmelcultures), Utrecht, the Netherlands

^a^ *ade^-^* auxotrophic for adenine; *asp^-^* auxotrophic for asparagine; *lys^-^* auxotrophic for lysine; *pro^-^* auxotrophic for proline; *try^-^* auxotrophic for tryptophane; *tyr^-^* auxotrophic for tyrosine.

**Supplementary Table 2 ǀ Accession numbers of ITS sequences**

| **Species** | **Strain/clone** | **Accession number** |
| --- | --- | --- |
| *M. andauensis* | 11-1120 (CBS 10809^T^) | Not deposited because of high heterogeneity |
|  | Clone Ian5 | KM243746 |
|  | Clone Ian6 | KM243747 |
|  | Clone Ian10 | KM243748 |
|  | Clone Ian42 | KM243743 |
|  | Clone Ian43 | KM243744 |
|  | Clone Ian51 | KM243745 |
| *M. fructicola* | 11-579 (CBS 8853^T^) | Not deposited because of high heterogeneity |
|  | Clone Ifr4 | KM213977 |
|  | Clone Ifr6 | KM213978 |
|  | Clone Ifrb17 | KM213982 |
|  | Clone Ifrb15 | KM213981 |
|  | Clone Ifrb13 | KM213980 |
|  | Clone Ifr9 | KM213979 |
|  | Clone Ifrc27 | KM213984 |
|  | Clone Ifrc26 | KM213983 |
